# Therapeutic role of PTEN in tissue regeneration for management of neurological disorders: stem cell behaviors to an in-depth review

**DOI:** 10.1038/s41419-024-06657-y

**Published:** 2024-04-16

**Authors:** Yue Li, Ruishuang Ma, Xia Hao

**Affiliations:** 1 State Key Laboratory of Quality Research in Chinese Medicine, Institute of Chinese Medical Sciences, University of Macau, 999078 Macau, China; 2 Department of Pharmaceutical Sciences, Faculty of Health Sciences, University of Macau, 999078 Macau, China; 3 State Key Laboratory of Component-Based Chinese Medicine, Institute of Traditional Chinese Medicine, Tianjin University of Traditional Chinese Medicine, 301617 Tianjin, China

**Keywords:** Neurological disorders, Stem cells

## Abstract

Phosphatase and tensin homolog deleted on chromosome 10 (PTEN) represents the initial tumor suppressor gene identified to possess phosphatase activity, governing various cellular processes including cell cycle regulation, migration, metabolic pathways, autophagy, oxidative stress response, and cellular senescence. Current evidence suggests that PTEN is critical for stem cell maintenance, self-renewal, migration, lineage commitment, and differentiation. Based on the latest available evidence, we provide a comprehensive overview of the mechanisms by which PTEN regulates activities of different stem cell populations and influences neurological disorders, encompassing autism, stroke, spinal cord injury, traumatic brain injury, Alzheimer’s disease and Parkinson’s disease. This review aims to elucidate the therapeutic impacts and mechanisms of PTEN in relation to neurogenesis or the stem cell niche across a range of neurological disorders, offering a foundation for innovative therapeutic approaches aimed at tissue repair and regeneration in neurological disorders.

**Figure:**
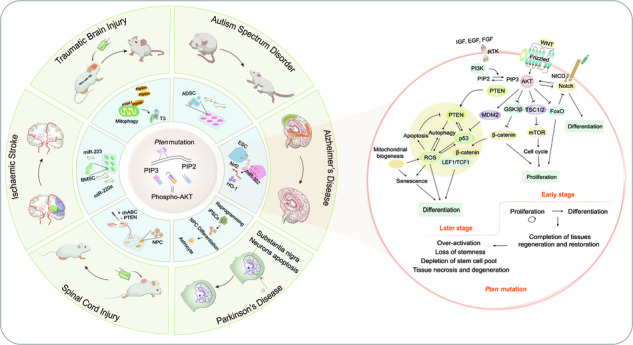
This review unravels novel therapeutic strategies for tissue restoration and regeneration in neurological disorders based on the regulatory mechanisms of PTEN on neurogenesis and the stem cell niche.

This review unravels novel therapeutic strategies for tissue restoration and regeneration in neurological disorders based on the regulatory mechanisms of PTEN on neurogenesis and the stem cell niche.

## Facts

1. PTEN plays a crucial role in stem cell self-renewal, proliferation, differentiation, survival and metabolism.
2. *Pten* gene expression is governed by various factors hinging on the cellular and organismal context.
3. PTEN directly or indirectly governs the expressions of a remarkably large number of genes and proteins.
4. Molecular mechanisms underlying the regulation of PTEN in fate determination of diverse stem cell populations, such as ESCs, iPSCs, NSCs, HSCs, MSCs, RPCs and tissue-derived adult stem cells, are still not fully illustrated yet.
5. PTEN participates in the management of various neurological disorders through coordinating signaling transduction.

## Open questions

1. How to control stem cell therapy effectively?
2. What are the consequences of excessive PTEN knockdown on the stem cell pool?
3. How to balance PTEN and its downstream signals and their wane and wax?
4. What are the specific roles of PTEN mutation at different stages of brain development?
5. How neurogenic niche cell and their cellular components influence the stem cell behaviors in the treatment of neurological disorders?
6. What ‘s the prospect of PTEN inhibitors in clinical application?

## Introduction

**Fig. 1:**
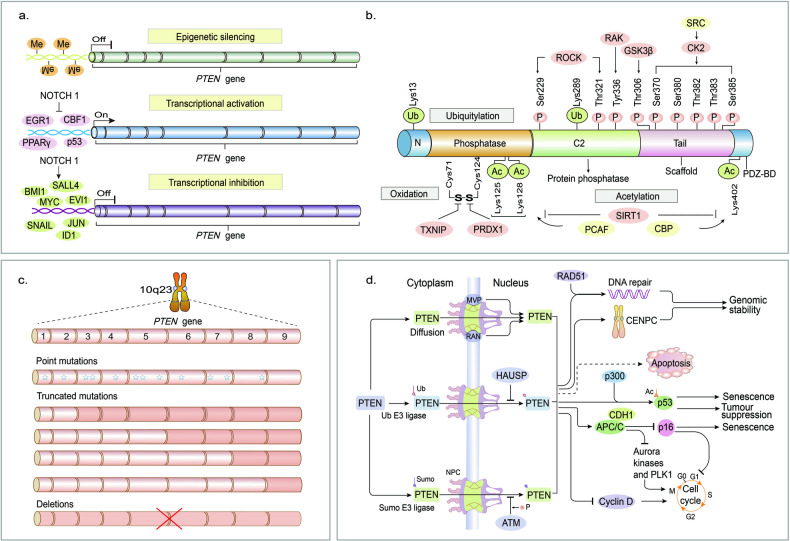
Structure, biological function, and mutation site of PTEN. **a** PTEN is regulated at various transcriptional levels. **b** PTEN post-translational modifications. **c** Genetic alterations of PTEN: Point mutations, truncated mutations, and allelic deletions causing PTEN inactivation or deletion. **d** In the nucleus, PTEN performs numerous cellular functions, mainly in a lipid-phosphatase-independent manner. Ac acetylation, APC/C anaphase-promoting complex, CDH1 CDC20-like protein 1, CENPC centromere protein C, NPC nuclear pore complex, PLK1 polo-like kinase 1, sumo sumoylation, Ub ubiquitylation.

*Pten* is a well-established tumor suppressor gene located on chromosome 10q23. The protein product of PTEN reportedly possesses lipid-phosphatase, protein-phosphatase, and phosphatase-independent activities [1]. It is widely acknowledged that PTEN functions depend on its lipid phosphatase activity by counteracting the phosphoinositide-3 kinase (PI3K)/protein Kinase B (AKT)/mTOR cascade by dephosphorylating phosphatidylinositol 3,4,5-triphosphate (PIP3) to phosphatidylinositol 4,5-bisphosphate (PIP2), associated with a plethora of cellular functions, including stem cell proliferation, differentiation, migration, genomic stability and even mitochondrial metabolism [2] (Fig. 1). PTEN is a powerful tumor suppressor [3], that plays a critical role in modulating the neurological system as well. Deletion of PTEN in mice results in symptoms such as seizures and autism spectrum disorder (ASD). Individuals with mutations in the PTEN gene have a higher risk of developing epilepsy [4] and autism [5].

Stem cells, including pluripotent stem cells, multipotent stem cells and unipotent stem cells, are involved in self-renewal and differentiation to maintain the terminal differentiation of the organism from early embryonic patterns to adult tissues. As for the broad scope of PTEN signaling, its regulatory role in cell proliferation, fate specification, energy metabolism and even cellular architecture in stem cells remains largely unclear. Therefore, an enhanced understanding of the contribution of biological processes, key signaling, and factors related to the stem cell niche will not only shed light on the processes that manage the functional integrity of the adult brain but also provide valuable insights into neurological illness. In this review, we mainly discuss the recent discoveries describing the mechanisms underlying the regulatory effect of PTEN on stem cell behavior and tissue restoration in physiological and pathological conditions.

## Pten and pluripotent stem cells

### PTEN and Embryonic stem cells

**Fig. 2:**
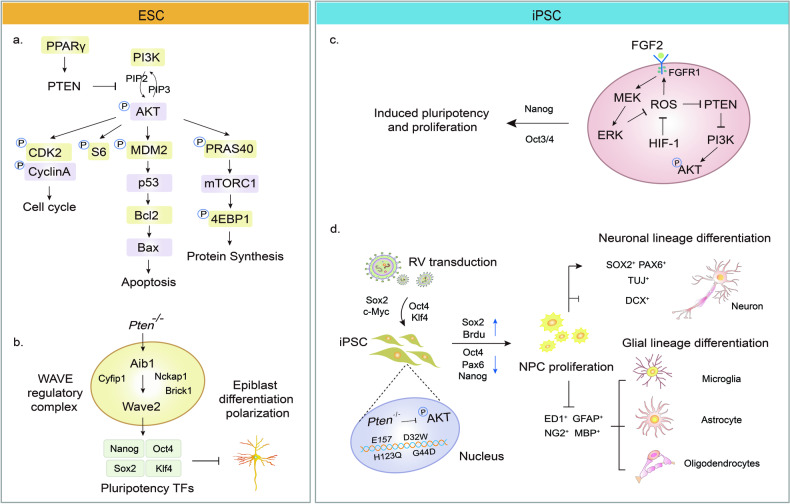
Representative scheme illustrating regulatory mechanisms of PTEN in ESCs and iPSCs. **a** PTEN dephosphorylates Abi1, negatively regulates WRC and induces differentiation and polarization of ESC into the ectodermal epithelium. **b** PPARy/PTEN/AKT mediates ESC proliferation and differentiation. **c** ROS/FGFR1/PAKT and ROS/PTEN/AKT promote iPSC production and maintenance of pluripotency. **d** PTEN knockdown in iPSCs can upregulate the expression of NPC-specific marker genes, concomitant with a decrease in the expression of neuronal lineage markers. WAVE WASP family Verprolin-homologous protein, CDK2 recombinant cyclin-dependent kinase 2, Bcl2 B-cell lymphoma-2, Bax Bcl2-Associated X, HIF-1 hypoxia-induced factor 1, Sox2 sex-determining region Y-box2.

Embryonic stem cells (ESCs) can self-renew and differentiate into any type of cells of the three embryonic germ layers in vivo and are distinct from adult stem cells. The main regulatory factors and signaling molecules in ESCs shape the microenvironment for self-renewal, differentiation and even apoptosis. The progression of PTEN deficient-ESCs into the mitotic S-phase, mediated by AKT/mTOR signaling or PPARγ/PTEN/PI3K/AKT survival pathway, leads to increased growth rate, proliferation and survival, but a spontaneous reduction in differentiation [6, 7] (Fig. 2a). PTEN deletion maintains the primitive pluripotency of ESCs by blocking glycogen synthase kinase-3β (GSK3β) activity, promoting the germline of ESCs to differentiate toward the ectodermal lineage [6, 8, 9] (Fig. 2b). Moreover, PTEN loss in ESCs can induce the formation of embryonic tumor cells. Considering that aggressive tumor cells have similar proliferation and plasticity properties to ESCs, PTEN regulation in the ESC microenvironment instead of the tumor microenvironment (TME) represents an ideal intervention to prevent early-stage tumorigenesis [10]. In addition, enhanced expressions of P53 and PTEN promote the expression levels of caspase 9 in human ESCs, but inhibit AKT phosphorylation and tumor growth, demonstrating that the PTEN-P53 signaling pathway governs cell cycle exit in tumors [11]. As a typical tumor suppressor gene, loss-of-function in PTEN can result in a deficiency of tumor suppressor signaling in the downstream cascades, providing a favorable environment for tumor growth. Apart from this, Abl-interactor 1(Abi1), a novel target of PTEN, has been identified as a key factor that induces embryoid body (EB) differentiation and polarization [8]. These findings suggest that PTEN deficiency accelerates cell cycle progression, induces AKT/mTOR signaling and promotes ESC self-renewal. The early blastocyst microenvironment can potentially inhibit cancer cell behavior by improving the TME. Increasing the quality and quantity of ESCs in co-culture systems can enhance this antitumor effect [10]. PTEN manipulation can help create a favorable ESC microenvironment, offering a potentially effective and safe cancer treatment option.

### PTEN and induced pluripotent stem cells

The ability of stem cells to renew and change can cause cancer in host tissues, especially with teratoma formation in human induced pluripotent stem cells (iPSCs) and hESC cell transplants. Targeted differentiation methods can lower the risk of teratoma. Although iPSCs closely resemble ESCs, they can be created by reprogramming somatic cells from different mammals and are a promising option for regenerative medicine. However, there is a risk of tumorigenicity from reprogramming, so it is important to ensure that oncogenic transgenes are not present in human iPSCs used for clinical cell therapy. The addition of specific TFs is a typical method to trigger reprogramming. For instance, in the absence of PTEN, the kruppel-like factor 4 (Klf4) and the sex-determining region of Y chromosome-box2 (Sox2) in the embryonic fibroblasts can accelerate the transformation rate to iPSC [12]. In the presence of PTEN, when fibroblast growth factor 2 (FGF2) is bound to its receptor, it promotes the mitogen-activated protein kinases (MAPK) signaling pathway, reduces reactive oxygen species (ROS) levels, and synergistically inhibits AKT phosphorylation [13] (Fig. 2c). Therefore, targeting PTEN can be used to induce iPSC differentiation towards endothelial cells [14], neuronal cells [15], and cerebral organoids (COs) [16].

The utilization of iPSC-derived neural cells presents a compelling alternative for studying neurological disorders stemming from physiological irregularities in PTEN. Neural precursor cells (NPCs) derived from iPSCs demonstrate rapid growth and morphological and immunohistochemical similarities to cortical neurons. These cells show enhanced capabilities in supporting neuronal survival and growth while blocking the proliferation and differentiation of astroglial cells [17]. Recent studies have demonstrated that PTEN knockdown can increase the expression of NPC-specific marker genes, including Sox2, T-domain transcription factor (Tbr2) and homeodomain-only protein X (Hopx), while simultaneously decreasing the expression of neuronal lineage markers such as β-Tubulin (Tuj1) and RNA binding protein Fox-1 homolog 3 (Rbfox3). This finding presents a potential solution to address the constraints of current neurogenesis studies [16] (Fig. 2d). Furthermore, the secretion of exosomes by iPSC-NPC has been shown to aid in the differentiation process towards neuronal lineages through the suppression of PTEN/AKT signaling or the enhancement of brain-derived neurotrophic factor (BDNF), ultimately contributing to the restoration of hypoxic and glucose-deficient neurons [18]. Consequently, the absence of PTEN is crucial in determining the neural differentiation trajectory of early iPSCs, resulting in heightened differentiation towards neuronal lineage cells while diminishing differentiation towards glial lineage cells. Moreover, the manipulation of PTEN or exogenous input of TFs to interact with PTEN has the potential to induce metabolic reprogramming in iPSCs and facilitate the generation of diverse cell and tissue types, thus serving as a crucial tool in the field of regenerative medicine. While heightened AKT signaling promotes a state of primordial pluripotency in iPSCs, the precise role of PTEN in regulating the balance between reprogramming and differentiation of various cell types and stage-specific lineages warrants further investigation.

## Pten and multipotent stem cells

### PTEN and Neural stem cells

**Fig. 3:**
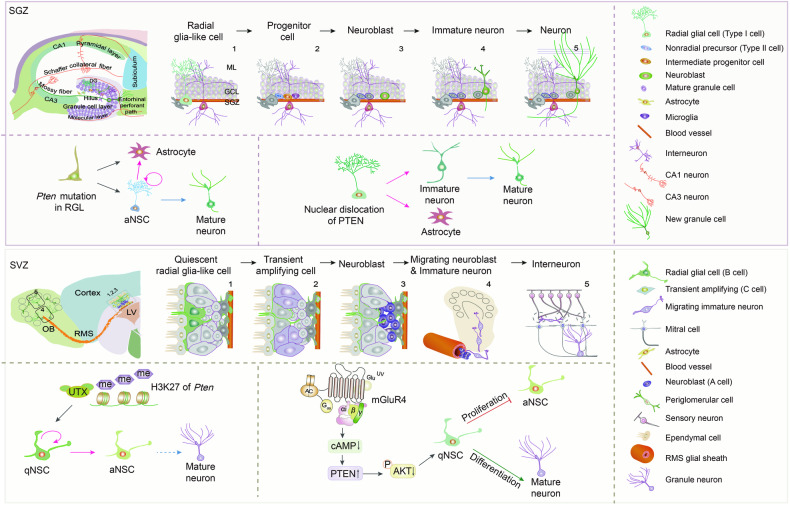
Schematic diagram of the effect of PTEN deletion on determining the fate of NSCs. The influence of PTEN on proliferation and differentiation of NSCs in SVZ and SGZ niches.

Neural stem cells (NSCs) in two specific regions of the adult brain, the subventricular zone (SVZ) and subgranular zone (SGZ) can give rise to newborn neurons, contributing to information processing associated with complex sensory and cognitive functions [19]. There is a rich literature available substantiating the role of PTEN in the ability of NSCs to differentiate into all neural lineages and to self-renew in these two regions of the adult brain. For instance, the loss of PTEN initially promotes the activation and self-renewal of hippocampal NSCs, but ultimately induces astroglial differentiation and NSC depletion within the stem cell pool [20]. Nuclear-specific deletion of PTEN in NSCs contributes to deficits in neuronal maturation, potentially participating in the pathogenesis of PTEN-autism spectrum disorder (ASD) [21]. Furthermore, premature loss of PTEN impedes the negative feedback of apoptosis and stress, impairs migration despite continued proliferation, and causes aberrant cellular morphology and function in newborn neurons [21] (Fig. 3). Notably, the epigenetic regulation of PTEN alters NSC proliferation and differentiation, consequently affecting neural regeneration. Moreover, direct upregulation of *Pten* promoter H3K27 trimethylation levels after ablation of UTX downregulates the protein and mRNA expression of PTEN, activates AKT/mTOR signaling, enhances paired box gene 6^+^ (Pax6^+^) NSCs in SVZ, and reduces the number of differentiated mature neurons in the cortex [22] (Fig. 3).

Lactate is the primary energy source for neurons to perform their physiological functions. It has been shown that PTEN deficiency in brain endothelial cells leads to aberrant adult neurogenesis due to sustained lactate accumulation, thereby hindering the terminal differentiation of hippocampal neurons [23]. This finding indicates that PTEN is crucial in regulating cellular energy and the metabolic environment during neurogenesis. Furthermore, metabotropic glutamate receptor 4 (mGluR4) activation could reduce intracellular cyclic adenosine monophosphate (cAMP) concentration and alleviate its inhibitory effect on PTEN, which in turn downregulates AKT phosphorylation, leading to reduced NSC proliferation, but enhanced neuronal differentiation in SVZ [24] (Fig. 3). Moreover, it has been shown that PTEN deletion can promote the conversion of NSCs into tumor stem-like cells by increasing the expression of Pax7, resulting in the development of a metabolic and gene expression profile associated with tumorigenesis [23]. Nevertheless, mitomycin C selectively triggers apoptosis in *Pten*^*-/-*^ NSCs and rescues the poor outcome caused by PTEN deficiency [25]. These findings suggest that transcriptional and post-translational modifications of PTEN are switches that initiate NSC self-renewal and differentiation, hence making it a vital biomolecule for treating psychiatric disorders [20, 21].

### PTEN and Hematopoietic stem cells

**Fig. 4:**
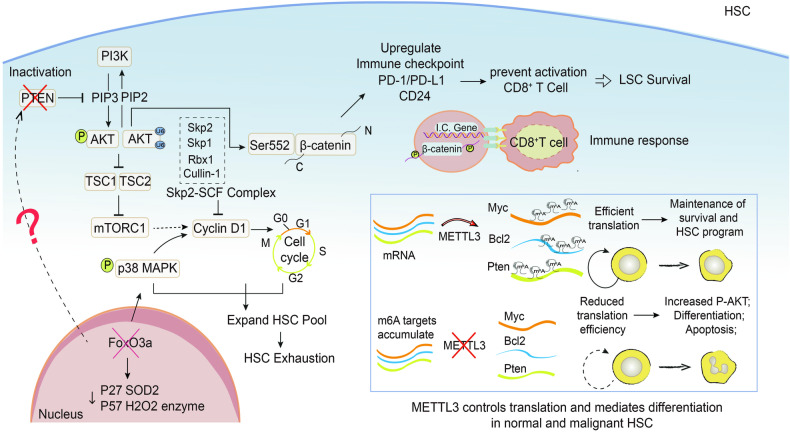
Schematic diagram of the effect of PTEN on the self-renewal of HSCs. PTEN deletion leads to alterations in downstream signaling molecules and post-transcriptional modifications in HSCs.

Hematopoietic stem cells (HSCs) give rise to blood cells of all lineages throughout life [26]. Intriguingly, it has been shown that PTEN can govern HSC mobilization and expansion in the spleen primarily via cell-autonomous mechanisms. PTEN deficiency not only influences HSCs response to inflammatory cytokines, including granulocyte colony-stimulating factor (G-CSF) and interferon-α (IFNα) [27] but also leads to HSC depletion and prevents these cells from stably reconstituting irradiated mice [28]. When AKT activation induces nuclear translocation of Forkhead Box O (FOXO) protein in the nucleus, it directly downregulates p27 and p57 levels, leading to excessive activation of HSCs, reduced number of colony-forming cells, and impairment of hematopoietic function [29]. Thus, FoxO3a, a downstream target of the PTEN/AKT pathway, is critical for HSC self-renewal and plays a pivotal role in maintaining the HSC pool [29]. Furthermore, loss of Skp2 not only increases HSC populations and long-term reconstitution ability but also rescues long-term reconstitution failure of HSCs on PTEN inactivation, indicating that Skp2 is a novel regulator for HSC quiescence and self-renewal [30] (Fig. 4). These demonstrate that PTEN has crucial roles in restricting the activation of HSCs, lineage fate specification, and leukemogenesis prevention [31].

The earliest feature in acute leukemia is the normal colonization of hematopoietic tissue but failure to mature and differentiate into blood cells by HSPCs [26]. This process is inevitably accompanied by alterations in epigenetic regulation [32, 33] When the m6A methyltransferase (METTL3) nucleotide modification is absent in HSCs, the effective translocations of PTEN (methylation), cellular-myelocytomatosis viral oncogene (c-Myc), and B-cell lymphoma-2 (BCL2) are downregulated, partially activating AKT, causing myeloid cells to differentiate towards a malignant phenotype and increasing apoptosis, providing a theoretical basis and direction for targeted therapy of myeloid leukemia [33]. Genomic destabilization by *Pten* knockdown in HSCs, with alterations in multiple downstream transcription factors, such as β-catenin and c-Myc overexpression, accelerates the self-renewal of leukemic stem cells (LSCs) [34]. Besides, the use of low doses of Doxorubicin to inhibit AKT-activated β-catenin in LSCs reportedly impedes immune checkpoint binding to the β-catenin promoter while mediating the synergistic activation of immune responses by extrinsic and intrinsic CD8^+^ cytotoxic lymphocyte (CD8^+^T cells), providing a novel strategy to overcome drug resistance and immune escape in cancer treatment [35] (Fig. 4).

The quiescent state of HSCs determines self-renewal after birth to provide hematopoiesis and reestablish the myeloid cell pool. PTEN/AKT is crucial to balance the activation and dormancy of HSCs [26]. Moreover, PTEN deficiency promotes malignant HSC differentiation and increases myeloid leukemia risk [31]. Importantly, HSCs lacking PTEN expression have been associated with cell cycle regulation and regenerative capacity defects, whereas AKT activation can inhibit FOXO protein expression, suggesting that FOXO targets can reverse the leukemia initiation program after PTEN deletion. In particular, PTEN plays a crucial role in regulating HSC activation, lineage, fate determination, and leukemia prevention, making it a promising option for cell transplantation and leukemia stem cell therapy [31, 33].

### PTEN and mesenchymal stem cells

Mesenchymal stem cells (MSCs) can differentiate into various cell types, including osteoblasts, chondrocytes, adipocytes, as well as bone marrow and dental pulp MSCs. MSCs represent a reliable option for stem cell therapy and clinical drug screening since they originate from different tissues [36, 37]. In this respect, using exosomes secreted by stem cells as natural carriers of drugs has become a research hotspot. MSC-derived exosomes loaded with *Pten* siRNA can repair spinal cord Injury by enhancing neuronal axon growth and vascularization [38]. MSC-derived exosomes have been demonstrated to promote diabetic wound healing by targeting PTEN/AKT pathways [39, 40]. Given that MSC-derived exosomes can migrate across the blood–brain barrier (BBB) to damaged areas in the brain, they broaden the therapeutic landscape for patients with CNS injury.

**Fig. 5:**
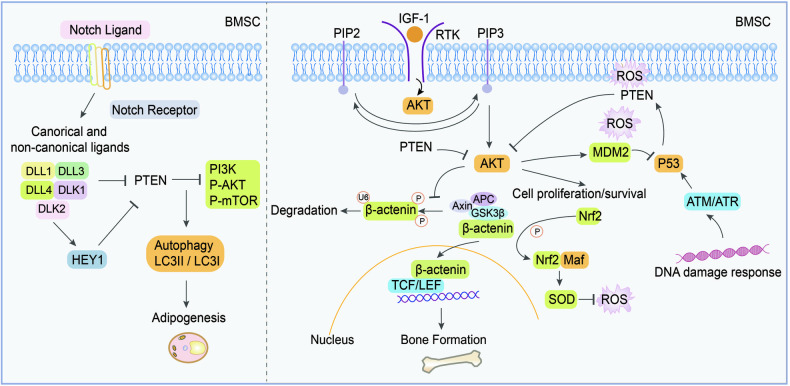
Representative scheme illustrating regulatory mechanisms of PTEN in BMSCs. Left, PTEN activation mediates anti-osteogenic differentiation by inhibiting AKT/GSK3β/β-catenin signaling pathway. Right, PTEN regulates the DNA damage response, oxidative stress, cell proliferation and survival in BMSCs. DLL human delta-like protein, DLK delta-like 1 homologue, LC3 microtubule-associated protein, RTK receptor tyrosine kinase.

*Bone marrow mesenchymal stem cells (BMSCs)* are adult stem cells of mesodermal origin with the potential for self-renewal and multidirectional differentiation to form bone, cartilage, adipose, and bone marrow hematopoietic tissue. Oxidative stress and inflammation severely affect the self-renewal and survival of BMSCs at concerned sites. Importantly, ROS inhibits BMSC proliferation, promotes differentiation into various cells and induces senescence [41]. It is well-established that the PTEN downstream effectors, AKT and P53, exhibit opposite regulatory functions to murine double minute2 (MDM2) during the crosstalk for transcriptional and post-translational modifications. Thus, virtual switches of life and death in BMSCs are initiated when the PTEN–AKT–P53–MDM2 closed-loop is formed [41]. Furthermore, when AKT is recruited at the plasma membrane, activating GSK3β/β-catenin signaling, BMSCs maintain bone mass as evidenced by a reduction in the level of PTEN phosphorylation but elevated protein and mRNA expression levels of PTEN [42]. Compared to dental pulp mesenchymal cells (DP-MSCs), BMSCs have been reported to exhibit a higher degree of *Pten* DNA methylation and more enrichment of the promoter repression tag histone 3 lysine 9 methylation *(*H3K9me2), accompanied by decreased PTEN level but an increased level of AKT phosphorylation. In comparison, DP-MSCs display higher osteogenic differentiation [43]. Besides, autophagy is involved in the lipogenic differentiation of BMSCs. Inhibition of Notch and upregulation of PTEN/PI3K/AKT/mTOR induces autophagy which mediates lipogenic differentiation of BMSCs, indicating that it is an essential pathological mechanism in lipid metabolic diseases [44] (Fig. 5). These findings indicate that PTEN has important roles in the occurrence and development of aging and metabolic diseases, regulating the self-renewal and fate specification of MSCs. Therefore, an enhanced understanding of molecular mechanisms underlying BMSC behaviors in response to pathological stimuli is helpful for future clinical applications in regenerative medicine.

*Adipose-derived stem cells (ADSCs)* are a well-recognized subset of MSCs with self-renewal and high migration capacity [45]. They have the potential to differentiate into endothelial cells, ectodermal and endodermal-derived cells to repair damaged, senescent tissues [46, 47]. Moreover, ADSCs secrete abundant exosomes and combine them with various chemokines, cytokines, miRNAs, and growth factors to enhance cytoprotection and improve the microenvironment, providing novel options for clinical research in regenerative medicine [46, 48]. For instance, miR-221/222 has been demonstrated to promote endothelial differentiation of ADSCs by modulating Pten/PI3K/Akt/mTOR signaling pathway. Furthermore, administration of miR-221/222 transfected ADSCs remarkably improves rat hindlimb ischemia and reduces inflammatory infiltration, providing a new therapeutic strategy in vascular formation and ischemic tissue regeneration [49].

The low survival rate of stem cells transplanted into the ischemic myocardium raises concerns about the effectiveness of stem cell therapy. Pretreatment of ADSCs with curcumin has been reported to reverse oxidative stress and promote angiogenesis through upregulating PTEN/AKT/P53 pathway and vascular endothelial growth factor (VEGF), respectively [48]. Transplantation of ADSCs pretreated with curcumin can significantly improve myocardial function and reduce apoptotic cells, suggesting that herbal pretreatment has huge prospects as an adjunct to stem cell transplantation [50].

ADSCs can potentially treat osteoporosis as they are sensitive to post-transcriptional modifications of PTEN and restore the balance of disrupted osteogenic and lipogenic differentiation [51]. Aberrant expressions of lncRNA-NEF and miR-155/PTEN have been documented in the serum of patients with osteoporosis. Accordingly, the overexpression of lncRNA-NEF regulates the miR-155/PTEN axis to inhibit adipogenesis and promote osteogenesis in ADSCs, emphasizing their potential to treat osteoporosis [51]. Interestingly, ADSCs can be converted into induced NSC-like cells and maintain neuronal lineage differentiation with a single transcription factor, Sox2, thus providing another alternative cell source for stem cell therapy for neurological diseases [52].

### PTEN and retinal progenitor cells

**Fig. 6:**
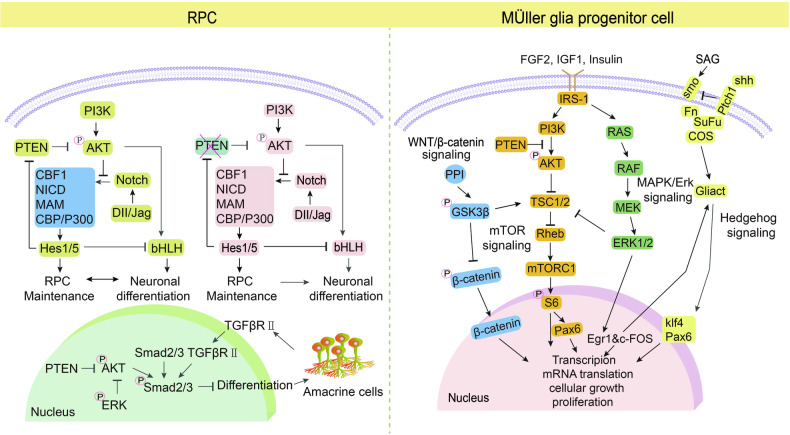
Representative scheme illustrating regulatory mechanisms of PTEN in RPCs. Left, PTEN promotes Notch signaling by inhibiting AKT activation, thereby preventing premature depletion of RPCs. Also, PTEN/ERK/TGFβ pathway triggers the differentiation of RPCs into amacrine cells. Right, PTEN is required for the proliferation of MGPCs by modulating Wnt/β-catenin and Hedgehog signaling pathways.

Retinal damage is a major cause of irreversible visual impairment and blindness. The limited regenerative capacity of the human retina makes it impossible to compensate for the lost cells, emphasizing the need for stem cell substitution [53]. Like HSCs, PTEN-deficient retinal progenitor cells (RPCs) undergo neurogenesis during early embryonic retinal development, resulting in premature attrition in the mature retina [54]. PTEN supports Notch signaling by negatively regulating AKT, promoting the formation of the Notch intracellular domain (NICD) TFs complex and preventing premature differentiation of RPCs. As a result of Hes family bHLH transcription factor 1 (Hes1)-induced inhibition of PTEN, AKT is reactivated to suppress Notch-initiated transcription, thus causing differentiation of RPC. When the Notch complex is disassembled, Hes1 fails to accumulate to repress PTEN expression, and re-expressed PTEN begins to repress AKT. This regulatory feedback loop coordinates retinal neurogenesis and RPC maintenance [55]. Accordingly, Notch transcriptional activator complex dissociation and the activation of mGluR4 relieve inhibition of PTEN and harmonize retinal neurogenesis and RPC retention [24, 55]. During the early embryonic development of the retina, RPCs gradually differentiate into amacrine cells, retina ganglion cells (RGCs), late progenitor cells, and mature MÜller cells [56]. Within the inner nuclear layer located beneath the retinal ganglion cells, PTEN facilitates the generation of amacrine cells by suppressing the PI3K/AKT pathway, Smad2/3 phosphorylation, and the modulation of the transforming growth factor beta (TGFβ) signaling pathway while also stimulating the activation of the MAPK signaling pathway. This suggests that PTEN serves as a favorable regulator in the differentiation of various subtypes of amacrine cells through the modulation of multiple downstream pathways, underscoring its multifaceted role as a mediator in the regulation of cell proliferation [57] (Fig. 6). Furthermore, mTOR signaling is required for the dedifferentiation of MÜller glial cells to acquire proliferative phenotypes. PTEN inhibition or elevated insulin-like growth factor1 (IGF1) activates mTOR signaling, which is required for reprogramming MÜller cells [54]. Recent study has explored the relationship between the mTOR signaling pathway and its involvement in activating the Wnt/β-catenin and Hedgehog (Hh) pathways, as well as inhibiting the glucocorticoid pathway [56] (Fig. 6). These findings indicate that the loss of PTEN may lead to excessive proliferation of RPCs by promoting AKT phosphorylation and suppressing Notch transcription, ultimately resulting in premature differentiation of RPCs. Indeed, PTEN refines our understanding of the intrinsic network complexity of adult stem cell proliferation and differentiation through interactions with multiple signaling pathways, such as PI3K/AKT/mTOR, MAPK, insulin, Wnt/β-catenin, and Hh pathways. The activation of human mature periodontal ligament stem cells within the retina leads to their differentiation into functional neurons and trans-differentiation into RGCs and RPCs by increasing the expressions of PTEN and VEGF. This suggests that trans-differentiation of human adult stem cells is the basis for the advancement of personalized medicine [58]. Furthermore, genetic deletion of PTEN and PTEN peptide promotes axonal regeneration in damaged optic nerves, improving functional recovery after nervous system injury [59, 60]. While PTEN is a known tumor suppressor, research has found that prolonged loss of PTEN in adult neurons can improve neuronal health [61]. Understanding how PTEN affects axonal regeneration after injury could have important clinical implications for treating and preventing nervous system diseases.

## Pten and unipotent stem cells

Tissue-specific adult stem cells are well-established to reshape local tissues throughout their life cycle. Herein we mainly focus on several populations of stem cells in which PTEN plays an important role in maintaining tissue renewal and wound reparation.

### PTEN and Hair follicle stem cells (HFSCs)

**Fig. 7:**
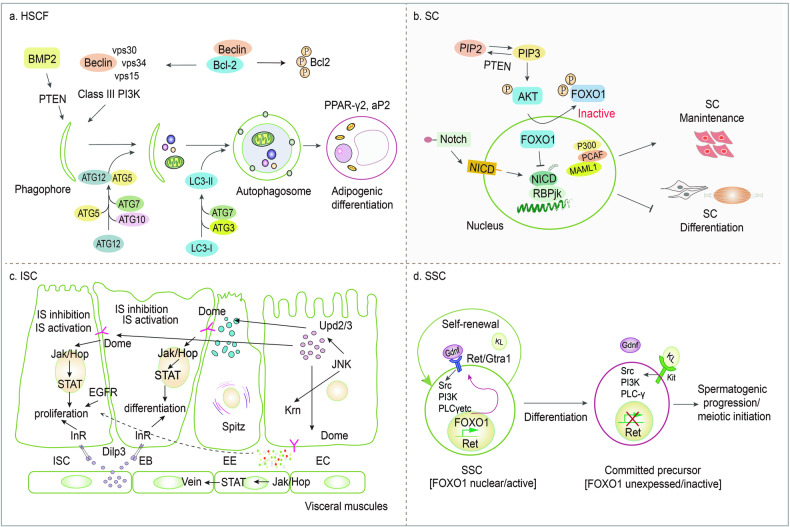
Representative scheme illustrating regulatory mechanisms of PTEN in HSCFs, SCs, ISCs and SSCs. **a** BMP2 upregulates PTEN expression and induces autophagy, leading to the enhanced differentiation of HSCFs. **b** Excessive increase in AKT/FOXO/Notch signaling disrupts the maintenance of SCs. **c** PTEN deletion activates IGF and promotes ISC proliferation and differentiation by upregulating the JAK/STAT signaling pathway. **d** The PI3K pathway rationalizes the self-renewal of SSCs via the control of Ret by FOXO1. Beclin myosin-like BCL2 interacting protein, Jak Janus kinase, InR insulin receptor, JNK c-Jun N-terminal kinase, Ret proto-oncogene, Src sarcoma gene.

PTEN and Hair follicle stem cells (HFSCs) exist in the outer root sheath of the hair follicle. They are pluripotent and self-renewing, differentiating to form smooth muscle cells, keratinocytes, melanocytes, and epidermal cells. Under oxidative stress, PTEN spontaneously translocates from the cytoplasm to the nucleus to initiate autophagy. Bone morphogenetic protein-2 (BMP2) induces autophagy and promotes the differentiation of HFSCs by upregulating PTEN expression to promote wound contraction and epidermal regeneration in skin-injured mice models [62] (Fig. 7a). Furthermore, systematic silencing and activation of HFSCs can induce cutaneous squamous cell carcinoma. PTEN deficiency promotes the massive proliferation of HFSCs and their offspring by upregulating the activation of AKT and β-catenin [63].

### PTEN and satellite cells (SCs)

PTEN and satellite cells (SCs) are myogenic stem cells that sustain skeletal muscle regeneration, tendon repair, and extracellular matrix formation through extensive self-renewal and multi-differentiation potential [64]. In response to damage and growth factors, SCs are activated and proliferate extensively. Most SC progenies undergo myogenic terminal differentiation and integrate to form new myotubes or fuse with damaged myofibers to repair the lesions. AKT is remarkably activated to induce FOXO1 cytoplasmic translocation of FOXO1 and suppression of Nothing signaling in *Pten*^*-/-*^ SCs, highlighting the important function of PTEN in the maintenance of SC quiescence via crosstalk with the Notch signaling pathway [64] (Fig. 7b).

### PTEN and intestinal stem cells (ISCs)

PTEN and intestinal stem cells (ISCs) are located at the base of the intestinal mucosal crypts and constitute the stem cell reservoir of the intestine. ISCs migrate from the basal layer to the intestinal lumen and form different intestinal mucosal cells [65]. In response to intestinal stress or injury, excess ISCs can initiate crypt formation and fission, recapitulating the production of crypt in fetal and neonatal intestines of PTEN-deficient mice [66]. PTEN deficiency often activates the cell cycle and promotes intestinal regeneration, leading to premature exhaustion [67]. IGF signaling in ISCs is involved in gene transcription for metabolism, intestinal homeostasis, and survival in Drosophila melanogaster [68]. The administration of a PTEN inhibitor to activate IGF has been shown to up-regulate the gene expressions of upd2 and upd3, which encode Janus kinase (JAK)-STAT and their target gene soc36. This process facilitates the proliferation and differentiation of ISCs into various types of intestinal cells for maturation by enhancing the expressions of the epidermal growth factor (EGF) ligand genes, *Spitz* and *Keren* [68] (Fig. 7c).

### PTEN and spermatogonial stem cells (SSCs)

PTEN and spermatogonial stem cells (SSCs)form pools of cells in different division states through self-renewal and differentiation necessary for spermatogenesis. PTEN deficiency disrupts the maintenance of the spermatogonial stem cell pool, leading to enlarged testes and depleted sperm counts after birth in mice [69]. Loss of PTEN inhibits the self-renewal and differentiation of SSCs, leading to over-activation of AKT and subsequent FOXO1 translocation, which suppresses downstream receptor protein tyrosine kinase-Ret signaling [70] (Fig. 7d). PTEN deficiency in the spermatogonial stem cell line results in a significant upregulation of Nanog expression, which negatively impacts spermatogenic potential and heightens the likelihood of teratoma formation [71]. These results suggest that PTEN plays a role in initiating cellular autophagy and facilitating the differentiation of somatic stem cells by phosphorylating AKT to deactivate FOXO protein and suppress Notch transcription. Hence, PTEN deficiency may facilitate the differentiation and maturation of intestinal stem cells by regulating insulin and JAK-STAT signaling pathways, thereby providing a novel therapeutic approach for tissue regeneration.

## Treatment of neurological disorders: Pten in stem cells

Neurological disorders encompass a diverse group of diseases in the central and peripheral nervous systems with limited treatment options and are the leading cause of disease burden globally. While stem cell therapy offers hope for patients suffering from brain injuries based on its potential to revive damaged nerves and the brain, the complexities of stem cell biology warrant further research to provide satisfactory data that support the rational, evidence-based application of these cells from a therapeutic perspective. PTEN regulates stem cell self-renewal and differentiation and coordinates signaling transduction among multiple stem cells in the stem cell niche to temporally and spatially ease the challenging task of treating the complex process of injury progression. Herein, we summarize the latest findings reported in the literature to highlight the therapeutic role of PTEN in stem cell behaviors for the management of neurological disorders such as autism, stroke, spinal cord injury, traumatic brain injury and neurodegenerative diseases, raising important questions to guide future studies and provide novel treatment options.

### Autism spectrum disorder

Autism spectrum disorder (ASD) is a neurodevelopmental disorder characterized by deficits in social behavior, restricted interests, and repetitive behaviors. Genetic, environmental, and other factors are widely thought to participate in the pathophysiology of this complex disorder. Nevertheless, the PTEN gene has been highly associated with autism [72]. Approximately 20% of patients with ASD have a PTEN mutation associated with giantism, epilepsy, and social impairment [73, 74].

Mutations in PTEN within the cortex or hippocampus of mice with ASD have been found to result in an increase in brain volume and mass. Furthermore, PTEN knockout in hippocampal NSCs leads to enlargement of the dentate gyrus (DG), disorganization of the granule cell layer, and the development of hypertrophic neurons with abnormal polarity [73]. Moreover, the differential gene expression of PTEN plays distinct roles in ASD. For instance, PTEN heterozygous mutations trigger the overgrowth of the mouse brain from birth to adulthood, induced by hyperplasia, which can be rescued by heterozygous mutation in Ctnnb1 (encoding β-catenin). These results highlight that the balance of PTEN and β-catenin signaling strictly governs normal brain growth trajectory, whereas disruptions in this equilibrium can potentially lead to aberrant brain growth [74]. In addition, human ADSCs transplantation has been demonstrated to alleviate autistic behaviors such as repetitive behaviors, impairments in social interaction and anxiety in ASD mice by restoring PTEN levels and rescuing the expressions of VEGF and interleukin 10 (IL-10). These results provide initial insights into the regulatory mechanisms of therapeutic effects of hADSCs in ASD [75] (Table 1).

**Table 1:** Therapeutic effects of PTEN-deficient stem cells on neurological disorders.

Disease	Treatment	Molecular mechanisms	Phenotypes	Reference
Autism spectrum Disorder	hADSC transplantation	PTEN/AKT	Social behavior; Angiogenesis	[70]
Ischemia/reperfusion	Electroacupuncture treatment	Notch/miR-223/PTEN	Neurogenesis	[76]
			Apoptosis	
	Exogenous addition of Tβ4	EGFR/Grb2/ERK1; EGFR/PI3K/AKT	NPC and OPC differentiation	[77]
			Apoptosis	
Spinal cord injury	NPC transplantation	N/A	Neural network connectivity	[79]
			Motor function	
	ADSC transplantation	ChABC and PTEN	Neuronal differentiation	[80]
			Gum scarring	
Traumatic brain injury	Thyroid hormone elevated	PINK1-mediated mitophagy	NSC differentiation	[83]
	ECPC-Exos transplantation	PTEN/PI3K/AKT	Tight junctions; Angiogenesis	[84]
	Acetylation-hMSCs transplantation	PTEN/PI3K/AKT/GSK3β	NSC proliferation	[94]
			Neuronal differentiation	
Parkinson’s disease	iPSC transplantation	PTEN/PI3K/AKT	Mitochondrial autophagy	[86]
		PINK1/Parkin		
	hiPSCs reprogramming	N/A	DN differentiation	[88, 89]
	PINK1	N/A	Astroglia differentiation	[87]
Alzheimer’s disease	ESC-specific secretion miR302	PTEN/PI3K/AKT/GSK3β	Oxidative stress	[92]
			Apoptosis	

### Ischemic stroke

Stroke is a severe neurovascular disease caused by focal or global interruption of blood supply to the brain, leading to transient or permanent neuropsychiatric impairments [76]. During this process, ATP levels are reduced, triggering lactate accumulation and activating the inflammatory cascade response, resulting in post-ischemic brain tissue necrosis [77, 78]. While autophagy may represent a critical route to cleaning damaged proteins or debris early in ischemic occurrence, excessive autophagy leads to substantial death [79]. It has been shown that stroke promotes NSC proliferation and migration of newly generated neuroblasts in the SVZ toward the ischemic boundary region and their differentiation into mature neurons. This suggests that endogenous neurogenesis is potentially one of the targets for rehabilitative therapy in patients with post-ischemic brain injury [80]. Recently, electroacupuncture (EA) therapy has been employed to treat the symptoms in an ischemic stroke rat model by inhibiting the PTEN signaling pathway, thereby causing NSC activation and apoptosis arrest to repair damaged tissue [81] (Table 1). Moreover, Thymosin beta 4 (Tβ4), a secreted 43 amino acid peptide, has been shown to promote NSC differentiation into oligodendroglia, associated with improved neurological outcomes in rat models of neurologic injury [82]. Additionally, the findings of both Tβ4 and PTEN-mediated oligodendrogenesis in NSCs may provide the basis for future treatment of stroke and other apoptosis-mediated diseases (Table 1).

### Spinal cord injury

Spinal cord injury (SCI) is a devastating traumatic condition associated with permanent neurological deficits. Growing evidence suggests that SCI encompasses a cascade of pathologic, biochemical and cellular processes, such as local spine deformation, electrolyte abnormalities, formation of free radicals, vascular ischemia, edema, posttraumatic inflammatory reaction, apoptosis or genetically programmed cell death. Microenvironment imbalance, defined as an increase in inhibitory factors and a decrease in promoting factors for tissues, including the imbalance in the differentiation of endogenous stem cells and the transformation phenotypes of microglia and macrophages, is the leading cause of poor SCI recovery [83]. Genetic deletion of PTEN at different times after spinal cord injury allows corticospinal tract (CST) neurons to regenerate axons and improves motor function recovery [84, 85]. Moreover, the identification of endogenous NSCs within the adult spinal cord has generated optimism for potential noninvasive therapies for SCI. This is due to the NSCs’ ability to produce new neurons that can integrate into existing neural circuits, facilitating the establishment of connections within neural networks and ultimately restoring motor function [86] (Table 1). Interestingly, the transplantation of PTEN-deficient ADSCs has been shown to improve the survival of transplanted cells, decrease glial scarring and promote functional recovery of SCI in rats [87] (Table 1). The benefits of genetic manipulation of *Pten* on ADSCs provide a potential strategy for stem cell therapy to treat SCI.

### Traumatic brain injury

Traumatic brain injury (TBI) is a leading cause of death and disability in individuals with trauma and continuous cognitive impairment, causing a decline in the quality of life [88]. TBI results in permanent cell loss and extensive neuronal death [89]. There is still no clinical treatment for rapid control and effective repair of TBI available. Furthermore, mitochondrial metabolism plays a role in the development of brain damage resulting from TBI. The PTEN-PINK1 signaling pathway has been identified as a mediator of the beneficial effects of Triiodothyronine (T3) on cell survival, mitophagy, and neurogenesis through neuron-NSC crosstalk, ultimately ameliorating neurological outcomes in mice subjected to TBI [90] (Table 1). Moreover, TBI results in the disruption of the blood-brain barrier (BBB). Endothelial colony-forming cells (ECFCs)-derived exosomes have been shown to promote the proliferation and migration of endothelial cells (ECs) following scratch injury in vivo, as well as improve BBB integrity and reduce brain edema in mice with TBI by modulating paracrine mechanisms and the PTEN signaling pathway [91] (Table 1). This finding suggests that in vivo delivery of ECFCs-derived exosomes with PTEN knockdown may provide a new approach for treating TBI. Importantly, stem cell transplantation represents a promising therapy for TBI. PTEN/AKT signaling pathway mediates the neuroprotective effects of hMSC engraftment in the hippocampus, accompanied by improved neurological outcomes and enhanced neurogenesis but reduced neural apoptosis and oxidative stress (Table 1). Taken together, transplantation of PTEN mutant pluripotent or somatic stem cells for neuronal rejuvenation represents a significant advancement in regenerative medicine, as it has been shown to stimulate neurogenesis and restore neurological dysfunction through epigenetic modification or exogenous supplementation of metabolic hormones to stem cells. The growth-promoting factors and signaling networks present in the stem cell niche, which are associated with tissue regeneration, play a crucial role in driving the neuronal differentiation of various stem cell populations. This highlights the potential of these stem cells to effectively treat TBI with complex pathophysiological stimuli.

### Parkinson’s disease

Parkinson’s disease (PD) is one of the most common movement disorders characterized by frequent psychiatric complications and a high prevalence of cognitive impairment. The Loss of dopaminergic neurons (DN) in the substantia nigra (SNpc), leading to the accumulation of alpha-synuclein (α-Syn) in Lewy vesicles, is the pathological hallmark of the disease [92]. Mitochondrial dysfunction is an influential initiator of this process, undermining the self-renewal of stem cells, especially after tissue damage. PINK1/Parkin-regulated mitophagy has been identified as an important pathway for adult neurogenesis and the removal of damaged mitochondria in PD patients [93]. The PTEN/AKT signaling pathway functions as an upstream regulator of PINK1 accumulation on damaged mitochondria, leading to reduced neuronal differentiation of NSCs. Additionally, this pathway plays a role in the regulation of endogenous PINK1-dependent mitophagy in neurons derived from human iPSCs [94–96] (Table 1). Therefore, a deeper comprehension of the relationship between sporadic Parkinson’s disease and mitochondrial dysfunction mechanisms will provide novel perspectives on stem cell therapy for dopamine cell replacement as an effective treatment for PD.

### Alzheimer’s disease

Alzheimer’s disease (AD) is a chronic, aging-associated neurodegenerative disorder characterized by progressive cognitive dysfunction due to microtubule-associated tau protein (Tau) hyperphosphorylation and amyloid-β (Aβ) plaque deposition in the brain [97]. No effective drug treatment is available to treat AD symptoms [98]. The ESC-specific miRNA-miR302 can protect against Aβ-induced neurotoxicity by inhibiting PTEN expression, leading to elevated expression of nuclear factor erythroid2-related factor 2 (Nrf2)/HO-1 expression. Additionally, miR302 suppresses insulin receptor substrate 1 (IRS-1) activation and GSK3β-mediated Tau protein hyperphosphorylation, ultimately attenuating Aβ-induced oxidative stress and apoptosis [99] (Table 1). This finding suggests that miR-302 can inhibit Aβ-induced cytotoxicity by inhibiting PTEN, thereby increasing Nrf2 levels and restoring insulin signaling. This underscores the potential application of miR-302 in stem cell therapy for the treatment of AD.

## Conclusion and perspectives

The works discussed in this review briefly illustrate the therapeutic impacts of PTEN on neurogenesis and various behaviors of different stem cell populations in neurological disorders, thus providing a unique novel perspective for their treatment. Given the diverse regulatory roles of PTEN in pluripotent and somatic stem cells, PTEN has huge prospects as a therapeutic target in regenerative medicine for the management of neurological disorders. Notably, the mechanistic workings of PTEN involve diverse signaling pathways in different neurological disorders; however, similar outcomes are achieved by regulating neurogenesis and stem cell niches. PTEN can target and modulate major intracellular signaling pathways associated with tissue restoration and regeneration. The ability of PTEN to target different underlying mechanisms highlights its potential as a therapeutic target for a wide range of neurological disorders.

The wide scope of action of PTEN extends to complex patterns of ectopic environment and tissue physiology. PTEN-dependent stem cells are spatially interconnected in neurogenic niches within specific tissues, preventing unauthorized stem cell depletion. Nevertheless, developmental plasticity may still exist. Early committed precursors receive pluripotent/somatic stem cells from the external environment in the neurogenic niche, allowing for the flexible transformation of their situation or identity into mature somatic cells. Furthermore, PTEN deletion in iPSCs, NSCs, RPCs, and MSCs has been shown to promote differentiation towards neuronal lineage while inhibiting differentiation towards the glial spectrum. The lineage gap between stem cells and progenitor cells status is not always strict in vivo and could be crossed during tissue destruction and reparation provided that the fates of stem and progenitor cells are interchangeable when the neurogenic niches come into contact. Recently, stem cells have been proven to acquire and accumulate functional epigenetic memories from different experiences they encounter, which is likely to have potential implications for tissue fitness and regenerative medicine [100]. Studying PTEN-regulated stem cells can help us understand PTEN-related disorders and the potential benefits of PTEN in regenerative medicine. An interdisciplinary approach is needed to uncover the molecular mechanisms of PTEN in response to different stimuli. Investigating specific stem cell types, developmental stages, and molecular factors influenced by PTEN is crucial for understanding how variations in this gene contribute to different traits and disorders in animal models. Further preclinical studies are needed to identify how specific PTEN variants lead to distinct phenotypes and to identify potential treatment targets. Understanding how PTEN regulates stem cells is crucial for diagnosing and treating neurological diseases. It also offers potential therapeutic strategies for cancer and neurological diseases, as well as opportunities for drug development. Thus, further studies focused on the cellular and molecular mechanisms underlying PTEN regulation of tissue stem cell response to physiological and pathological stimuli would help us take the therapeutic promise of PTEN in tissue regeneration closer to its potential. Finally, a deeper analysis of regulatory mechanisms of PTEN with regards to neurogenesis or stem cell niche will inspire novel approaches to better understand the integrative capacity of the adult brain and further refine current knowledge on the generative and regenerative potential of the niche not only as active participants in pathophysiological events but as pharmacological targets or even as therapeutics for neurological illness.

## Data Availability

There are no experimental datasets given that this is a review article that is prepared based on a literature review.
